# Changes in Spinal and Corticospinal Excitability in Patients with Chronic Ankle Instability: A Systematic Review with Meta-Analysis

**DOI:** 10.3390/jcm8071037

**Published:** 2019-07-16

**Authors:** Kyung-Min Kim, Joo-Sung Kim, David Cruz-Díaz, Seungho Ryu, Minsoo Kang, Wolfgang Taube

**Affiliations:** 1 Department of Kinesiology and Sport Sciences, University of Miami, Coral Gables, FL 33146, USA; 2 Department of Health Sciences, Faculty of Health Sciences, University of Jaén, 23071 Jaén, Spain; 3 Health and Sport Analytics Laboratory, Department of Health, Exercise Science and Recreation Management, The University of Mississippi, University, MS 38677, USA; 4 Department of Neurosciences and Movement Sciences, University of Fribourg, 1700 Fribourg, Switzerland

**Keywords:** Hoffmann reflex, transcranial magnetic stimulation, arthrogenic muscle inhibition, arthrogenic muscle response, neural adaptation, ankle sprain, functional ankle instability

## Abstract

The objective of this systematic review with meta-analysis was to determine alterations in spinal and corticospinal excitability of ankle muscles in patients with chronic ankle instability (CAI) compared to uninjured controls. Independent researchers performed comprehensive literature searches of electronic databases and included studies that compared groups with and without CAI and investigated neural excitability with Hoffmann reflex (H-reflex) and/or transcranial magnetic stimulation (TMS). A fixed-effect meta-analysis was conducted to determine group differences for (1) soleus and fibularis maximal H-reflex (Hmax)/maximal M-wave (Mmax)-ratios, and (2) soleus and fibularis longus cortical motor thresholds (CMTs). Seventeen studies were included in the current meta-analysis. They showed that the Hmax/Mmax-ratios of the soleus and the fibularis longus in the CAI group were significantly lower than those in the uninjured control group (soleus: d = −0.41, *p* < 0.001; fibularis longus: d = −0.27, *p* = 0.04). There was no evidence for changes in the CMT. This systematic review is the first to demonstrate evidence that patients with CAI present decreased spinal reflex excitability in the soleus and fibularis longus. However, there is no evidence of changes in supraspinal excitability when considering only the CMT. The latter result needs to be interpreted with caution as all except one study demonstrate some changes at the supraspinal level with CAI.

## 1. Introduction

Chronic ankle instability (CAI) is one of the most prevalent debilitating conditions in athletic populations [1,2]. A high percentage (23% to 61%) of athletes are identified as having CAI, with soccer, basketball, and volleyball being the most represented team sports [3,4,5,6]. In addition, recent research has highlighted that CAI is a public healthcare burden affecting not only athletic but also general populations [1]. CAI is often characterized by feelings of unstable ankles, frequent episodes of the ankle giving way, prolonged symptoms, and/or recurrent ankle injuries [2]. There is growing evidence that CAI significantly affects patient-oriented outcomes such as physical activity level [7,8] and health-related quality of life [9]. More clinically concerning, there is emerging evidence that CAI may be the primary source of early onset of post-traumatic osteoarthritis [10,11,12,13]. Despite research efforts made over the past six decades, the etiology of CAI remains unclear.

CAI has been traditionally thought to be attributed to mechanical and functional insufficiencies [14]. However, recent research has shown that mechanical inadequacies (i.e., pathological joint laxity) are not consistently presented in individuals with CAI although they are the common consequences of initial ankle sprains [1,2]. Proprioceptive deficits arising from a loss of mechanoreceptors in the sprained ankle joint have often been cited to explain the underlying mechanism of sensorimotor impairment found in CAI studies [15]. The sensory deficits have provided significant insights into the etiology of CAI and led to the development of effective therapeutic approaches (i.e., proprioceptive training) [16,17]. However, more recently, this feedback-based mechanism has been challenged because on its own it is unable to explain all phenomena that are associated with CAI [18,19,20,21,22,23,24,25,26]. For example, patients with unilateral CAI were shown to suffer from bilateral deficits in single-limb balance [22]. In addition, there is altered sensorimotor control in joints proximal to the CAI-involved ankle joint during various motor tasks including balance [18,24], gait [20,23], and drop landing [19,26]. Patients with CAI also presented neuromuscular deficits of the lower extremity before heel contact with the ground during gait and before landing [21,23,25]. These observations suggest that CAI is not only related to sensory deficits but is also associated with changes in the efferent motor control.

The efferent control of motor tasks greatly relies on the activity of supraspinal motor centers and of spinal reflex circuitries. One way to assess this activity is by measuring neural excitability at the spinal and/or motor cortical levels (e.g., the primary motor cortex). Research into neural excitability in patients with CAI has grown substantially over the past decade. The earliest studies [27,28] investigated the spinal excitability of lower leg muscles using the electrically evoked Hoffmann (H-) reflex and found decreased spinal excitability in the soleus and fibularis longus muscles that may limit optimal muscle activation vital for joint stability. Subsequent studies [29,30,31,32] attempted to relate these neurophysiological changes to clinical or functional outcomes and implied that the decreased neural drive to the spinal motoneurons innervating ankle stabilizers may contribute to sensorimotor deficits, which in turn may lead to functional limitations and self-reported disability. In addition to decreased spinal excitability, recent research has begun to examine supraspinal excitability in patients with CAI using transcranial magnetic stimulation (TMS). Multiple TMS outcomes (e.g., cortical motor threshold, motor evoked potential (MEP), corticomotor output map) have indicated that CAI may be related to sensorimotor reorganization in the brain and/or changes within descending pathways to the alpha motoneurons (i.e., the corticospinal tract) [33,34,35,36]. Collectively, the alterations in neural excitability at both spinal and supra-spinal levels suggest that neuroplastic adaptations appear to exist and may contribute to the sensorimotor dysfunction commonly seen in patients with CAI [37]. It is noted, however, that some studies [32,38,39,40] failed to identify neural changes in CAI patients. These conflicting results across individual studies may be due to small sample sizes, which raises the need for a synthesis of the data to provide conclusive evidence. Therefore, the purpose of this systematic review with meta-analysis was to determine alterations in spinal and corticospinal excitability of ankle muscles in patients with CAI compared to uninjured controls without a history of ankle sprain. This investigation is critical to providing greater insights into neurophysiological mechanisms underlying sensorimotor control deficits associated with CAI, which will enhance the current understanding of the most prevalent condition in the physically active.

## 2. Experimental Section

The current systematic review with meta-analysis was conducted according to the guidelines listed in The Preferred Reporting Items for Systematic Reviews and Meta-Analysis Statement (PRISMA) [41].

### 2.1. Eligibility Criteria

Studies were considered eligible for inclusion if they had at least one group with CAI and one without CAI (uninjured control group) and investigated neural excitability with the H-reflex and/or transcranial magnetic stimulation (TMS). The electrically evoked H-reflex, which is an analog to the stretch reflex but bypasses the muscle spindles, allows assessment of spinal reflex excitability [42]. TMS is a non-invasive technique to assess corticospinal excitability by generating a magnetic field over the targeted brain area where cortical cells are electrically activated [43]. Studies were excluded if they (1) recruited patients with acute ankle sprains or other ankle and foot pathologies, (2) examined neural function with electroencephalography or used other neurophysiological measures (i.e., stretch reflex), (3) were published in a non-English language, or (4) were reported as abstracts due to the risk of duplicating data.

### 2.2. Literature Search Strategy

We performed comprehensive literature searches to identify peer-reviewed journal articles on the neural excitability of ankle muscles in patients with CAI. Electronic databases (Web of Science, PubMed, CINAHL, and SPORTDiscus) were searched from their inception until April 2019. Two librarians with expertise in developing a search strategy in the medical and sports science fields were consulted to assist in developing comprehensive search strategies. Two primary investigators (K.M.K and J.S.K) then established search terms using keyword searching and Medical Subject Headings (MeSH) vocabulary. The search strategies were applied using various combinations of the following key terms: (“ankle sprain” OR ‘joint instability” OR “functional ankle instability” OR “chronic ankle instability” OR “unstable ankle”) and (“neural excitability” OR “supraspinal excitability” OR “corticospinal excitability” OR “motor neuron pool excitability” OR “motor evoked potential” OR “motor threshold” OR “transcranial magnetic stimulation” OR “TMS” OR “spinal excitability” OR “spinal reflex activity” OR “Hoffman reflex” OR “H-reflex” OR “arthrogenic muscle response” OR “arthrogenic muscle inhibition”). In addition to database searches, we performed manual searches of the reference lists of relevant studies.

### 2.3. Study Selection and Data Extraction

All articles retrieved through database searches were assessed for eligibility after duplicates were identified and excluded. Two independent reviewers (K.M.K and J.S.K) screened the titles and abstracts of all the articles identified from database searches against the selection criteria, with all authors involved in resolving any conflicts. The two reviewers then further screened the full text of all remaining articles and discussed any discrepancies to reach a consensus. Following determination of the studies to be included in the review, the reviewers independently extracted the following data from each included study: author, publication year, study design, inclusion criteria for CAI (experimental) and comparison (control) groups, participant characteristics, stimulation settings, and specific neural excitability outcomes. The reviewers extracted the means and standard deviations of all neural excitability outcomes from each study as well as sample sizes of the CAI and comparison groups. Authors were contacted where data were not reported in the articles [44]. The extracted data were cross-checked to verify their accuracy and any disagreements between reviewers were resolved through discussion.

### 2.4. Assessment of Methodological Quality

Two primary reviewers (K.M.K and J.S.K) independently assessed the methodological quality of each included study using the modified version of the Quality Index instrument, initially developed by Downs and Black [45]. The original Quality Index, consisting of 27 items, has been modified to include only 14 items to enhance its utility. This modified version has been commonly used in the meta-analysis studies on foot and ankle injuries [46,47,48,49]. For the evaluation, each item can be scored with 1 point upon a “Yes” response, except for one item regarding the distribution of principal confounders that can be scored with 2 points upon a “Yes” response or 1 point with a “partially” response. Thus, a total of 15 points is the possible maximum score. Subsequently, the points are expressed as percentage values (points obtained divided by 15 points and multiplied by 100). The following guideline was used to determine the level of methodological quality: 0%–59% was considered as poor, 60%–74% as moderate, and 75%–100% as high quality [49].

### 2.5. Data Analysis

We utilized the Comprehensive Meta-Analysis software (Version 3.0, Biostat, Inc., Tampa, FL, USA) to analyze and perform a meta-analysis to determine differences in neural excitability between groups with and without CAI using a fixed-effect model. Effect size estimates using Cohen’s d were employed to quantify the group differences. In accordance with Cohen’s guideline, the magnitude of the effect size was interpreted as follows: d = 0.2 small, d = 0.5 medium, and d = 0.8 large [50]. Between-study heterogeneity was determined with Cochran’s Q statistic and the I^2^ index. In accordance with Higgins’ guideline, the I^2^ index was interpreted as follows: >25% low, >50% moderate, and >75% high heterogeneity [51]. Finally, Egger’s test was performed to examine the risk of bias across studies. The level of significance for all statistical analyses was set a priori at *p* ≤ 0.05.

## 3. Results

### 3.1. Search Findings

**Figure 1:**
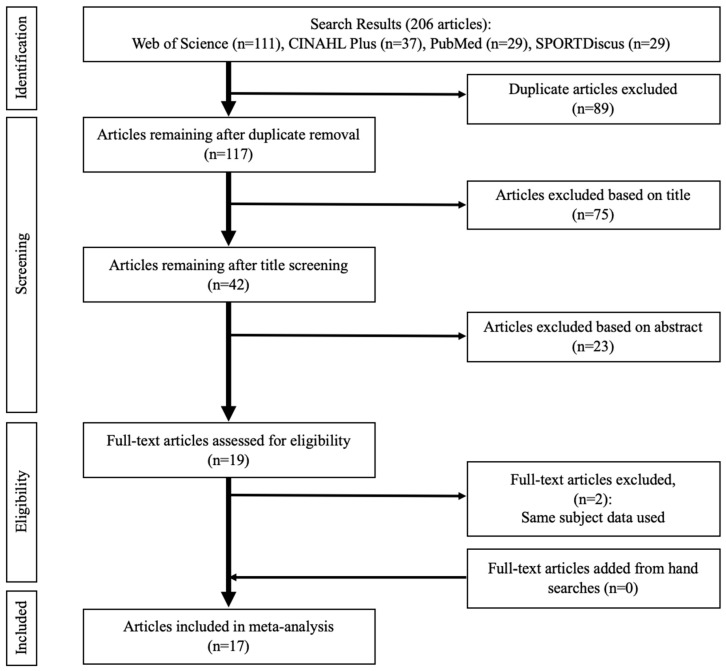
PRISMA flow diagram illustrating different stages of study screening and selection.

Figure 1 illustrates the search findings. The initial searches through the electronic databases identified a total of 206 studies. After removing 89 duplicate studies, 117 studies remained, and their titles were screened, resulting in 75 studies being excluded. Abstracts of the remaining 42 studies were further screened, causing an additional 26 studies to be excluded. The full-text screening performed over the remaining 19 studies determined 17 studies for final inclusion [27,28,32,33,34,35,36,38,39,40,44,52,53,54,55,56,57], with two studies [31,58] excluded because they reported the same subject data as two other studies that were already included [52,53]. No additional studies were identified based on the references in previously included studies. Table 1 details the characteristics of the included studies.

### 3.2. Methodological Quality

Two reviewers (K.M.K and J.S.K) independently scored a total of 238 methodological quality items (14 items × 17 studies) and initially agreed on 228 items (96%). Cohen’s kappa inter-rater reliability analysis demonstrated almost perfect agreement between the two reviewers (K = 0.915, 95% confidence interval of 0.860–0.970). Final consensus for all items (100%) was achieved upon further discussion. Table 2 shows the quality index scores of individual studies. The average score (65.9 ± 7.4%) indicated that overall, study quality was moderate. Fourteen studies (82.4%) clearly described study purposes, primary outcomes, main findings, estimates of random variability, and actual probability values. However, only eight studies (47.1%) explicitly described participant characteristics. While most studies were clear about reporting information about data dredging, statistical tests, the reliability and validity of outcome measures, and the participant recruitment pool, no studies reported information that helped to determine if participants were representative of the entire population and if participants were recruited over the same time period.

### 3.3. Study Characteristics

#### 3.3.1. Study Design

All 17 studies [27,28,32,33,34,35,36,38,39,40,44,52,53,54,55,56,57] utilized a case-control study design that had an experimental group with CAI and a comparison group consisting of age-matched persons who never sprained their ankles (uninjured controls). Three studies [32,55,57] had an additional comparison group consisting of participants who sprained their ankles once but did not suffer from any symptoms associated with CAI (copers). In addition, four studies [38,39,52,56] employed a cross-over design to determine the effectiveness of an intervention on neural excitability outcomes in both the CAI and uninjured control groups.

#### 3.3.2. Participants 

A total of 643 participants were enrolled in the included studies, with 311 participants with CAI, 272 uninjured controls, and 60 copers. Inclusion criteria for CAI were similar across studies including at least one previous ankle sprain, current feelings of ankle instability and/or episodes of the ankle giving way, and self-reported ankle dysfunction. Uninjured controls or copers were similarly recruited and were matched with the CAI group by age, height, and weight. All participants were young adults, with group mean ages ranging from 19.8 to 26.5 years and with 259 males and 384 females. 

#### 3.3.3. Outcome Measures

All 17 studies primarily investigated neural excitability in patients with CAI using peripheral nerve stimulation (PNS) to elicit H-reflexes and/or TMS to evoke MEPs: there were twelve PNS studies [27,28,32,38,39,44,52,53,54,55,56,57], three TMS studies [35,36,40], and two studies [33,34] that used both techniques. The technique for eliciting H-reflexes appeared to be consistent across all studies, involving 1 ms square-wave pulses that were delivered 10 to 20 s apart to prevent post-activation depression. All H-reflex studies reported Hmax/Mmax ratios as an outcome for estimating “spinal reflex excitability”. For the TMS technique, almost all studies (four out of five) used the double-cone coil to generate magnetic stimuli up to 1 or 1. 4 Tesla that were delivered 10 to 15 s apart, except for one study [40] which utilized the figure-8 coil to deliver magnetic stimuli 5 s apart. All five TMS studies reported cortical motor threshold (CMT) as the primary outcome for quantifying corticospinal excitability. However, additional TMS parameters were used including MEP [33,34,36], cortical silent period [36,40], and/or corticomotor map parameters [33]. 

All TMS studies, and most PNS studies, collected data when participants were either in a lying position or were seated. Only three studies assessed “spinal excitability” (H-reflexes) while standing [32,53,54].

### 3.4. Neural Excitability Meta-Analysis

Only one outcome measure per study is recommended to avoid violation of the assumption of independence in the meta-analysis [51]. Standard practice is to choose a common neural excitability measure in a study reporting multiple measures. For spinal excitability, an outcome measure of Hmax/Mmax ratio at rest (i.e., lying or sitting) was selected because it was commonly reported in 11 out of 14 studies that used H-reflex. The other three studies [32,53,54] reported the same measure, but in the bipedal stance condition; therefore, bipedal Hmax/Mmax ratios were selected. For cross-over H-reflex studies [38,39,52] reporting two baseline measures on different days, random selection was used to include one of the baseline measurements. Furthermore, one H-reflex study [57] had three subgroups with CAI: (1) perceived instability, (2) recurrent ankle sprains, and (3) a combination of both. The group with perceived instability and recurrent ankle sprains was selected because CAI participants in other studies reported both perceived instability and recurrent ankle sprains. For supraspinal excitability, a measure of cortical motor threshold (CMT) was selected because it was used in all five TMS studies. Regarding the target muscles, several lower extremity muscles were tested but all included studies examined either the soleus [19,32,38,53,54,55,56,57], the fibularis longus [28,33,34,35,39], or both [27,40,44,52]. Thus, the current study performed a meta-analysis to determine the difference between the CAI and uninjured control groups for each outcome: (1) soleus Hmax/Mmax ratio, (2) fibularis longus Hmax/Mmax ratio, (3) soleus CMT, and (4) fibularis longus CMT. A negative effect size of the Hmax/Hmax ratio represents decreased spinal reflex excitability while a positive effect size of CMT reflects decreased corticospinal excitability. 

#### 3.4.1. Soleus Hmax/Mmax Ratio

**Figure 2:**
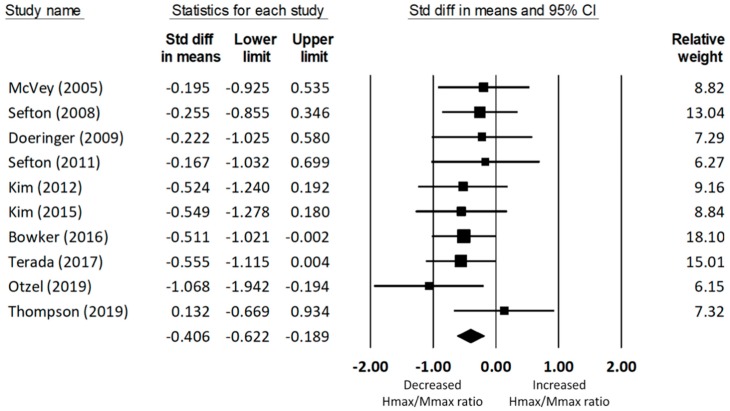
Forest plot illustrating Cohen’s d effect sizes for the soleus Hmax/Mmax ratio between groups with and without CAI and their 95% confidence intervals.

A total of ten studies [27,32,38,44,52,53,54,55,56,57] on soleus spinal excitability produced data for 292 (154 CAI and 138 uninjured control) participants. The meta-analysis concluded that the Hmax/Mmax ratio of the soleus was significantly reduced in the CAI group compared to that of the uninjured control group (d = −0.41, 95% CI = −0.62 to −0.19, *p* < 0.001; see Figure 2). The effect size of this group difference was small to medium. There was no significant heterogeneity associated with the observed effect (Q(9) = 5.69, *p* = 0.77, I2 = 0%). 

#### 3.4.2. Fibular Longus Hmax/Mmax Ratio

**Figure 3:**
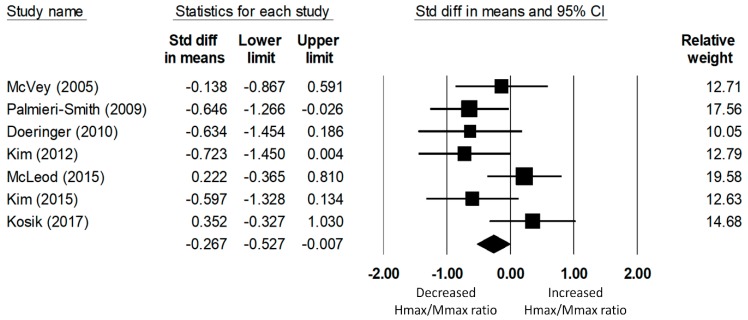
Forest plot illustrating Cohen’s d effect sizes for the fibular longus Hmax/Mmax ratio between groups with and without CAI and their 95% confidence intervals.

A total of seven studies on fibularis longus spinal excitability yielded data for 235 (118 CAI and 117 uninjured control) participants. The meta-analysis determined that the Hmax/Mmax ratio of the fibularis longus was significantly reduced in the CAI group compared to that of the uninjured control group (d = −0.27, 95% CI = −0.53 to −0.01, *p* = 0.04; see Figure 3). The effect size of this reduction was small to medium. The heterogeneity of the effect sizes was not significant (Q(6) = 10.47, *p* = 0.11, I2 = 42.71%). 

#### 3.4.3. Soleus Cortical Motor Threshold

**Figure 4:**
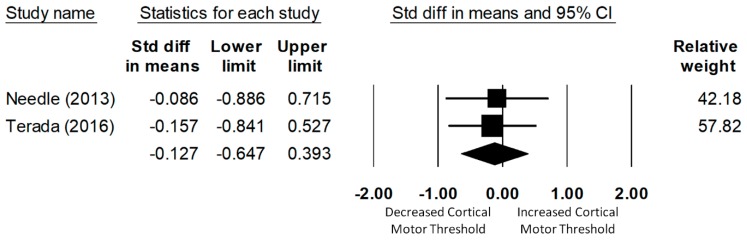
Forest plot illustrating Cohen’s d effect sizes for the soleus cortical motor threshold between groups with and without CAI and their 95% confidence intervals.

Only two studies examined the corticospinal excitability of the soleus. The meta-analysis of data for 57 (28 CAI and 29 uninjured control) participants found that CMT did not significantly differ between the CAI and the uninjured control group (d = −0.13, 95% CI = −0.65 to 0.39, *p* = 0.63), as shown in Figure 4. Significant heterogeneity of the effect sizes was not found (Q(1) = 0.02, *p* = 0.89, I2 = 0%). 

#### 3.4.4. Fibularis Longus Cortical Motor Threshold

**Figure 5:**
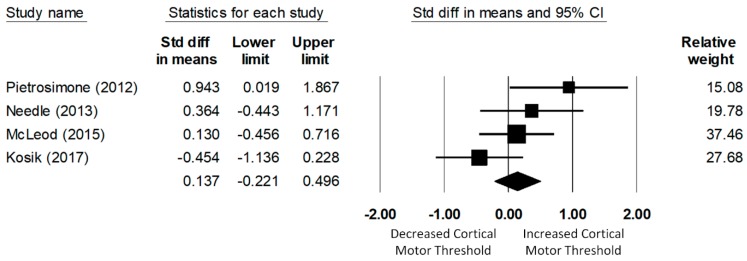
Forest plot illustrating Cohen’s d effect sizes for the fibular longus cortical motor threshold between groups with and without CAI and their 95% confidence intervals.

A total of four studies investigated the corticospinal excitability of the fibularis longus. The meta-analysis of data of 123 (61 CAI and 62 uninjured control) participants determined that the CMT of the fibularis longus in the CAI group was not significantly different from that of the uninjured control group (d = −0.14, 95% CI = −0.22 to 0.50, *p* = 0.45; see Figure 5). The heterogeneity of the effect sizes was not significant (Q(3) = 6.11, *p* = 0.11, I2 = 51%). 

### 3.5. Risk of Bias across Studies

Egger’s regression test was performed to examine the risk of bias across studies for each meta-analysis. Applying this test, we found no significant publication bias for any outcome: soleus Hmax/Mmax ratio (intercept = 0.71, *p* = 0.64), fibularis longus Hmax/Mmax ratio (intercept = −5.40, *p* = 0.30), and fibularis longus CMT (intercept = 4.70, *p* = 0.36). Egger’s test was not performed for the soleus CMT due to the small number of studies (*n* = 2).

## 4. Discussion

The current systematic review with meta-analysis is the first to synthesize data from individual studies investigating the neural excitability of lower extremity muscles in patients with CAI. This review found moderate-quality evidence indicating decreased spinal reflex excitability of the soleus and fibularis longus muscles in young adults with CAI when compared to age-matched controls without a history of ankle sprain. These findings appear to be in line with a growing body of evidence showing that neural adaptations occur in other musculoskeletal conditions [37,59,60] and support the emerging therapeutic strategies addressing neuroplasticity in the field of sports medicine [61,62,63,64]. However, we did not see a group difference in corticospinal excitability of either muscle between CAI and healthy control subjects. The lack of group differences in corticospinal excitability needs to be interpreted with caution for the reasons discussed in the following sections. 

### 4.1. Spinal Reflex Excitability Associated with CAI

The small-to-medium effect size of the group difference in the soleus Hmax/Mmax ratio found in the present review is somewhat consistent with individual studies, except for the latest two studies [32,56]. The group difference in the fibularis longus Hmax/Mmax ratio was of small-to-medium effect size. However, individual studies reported varying effect sizes. Collectively, these results imply that the spinal reflex excitability of ankle muscles was significantly decreased in patients with CAI. Reflex inhibition has also been observed in other musculoskeletal conditions such as chronic low back pain [65], and hip [66] and knee injuries [67,68]. 

#### 4.1.1. Functional Consequences of Reflex Inhibition in CAI Patients

Sefton et al. [58] found that when considering performance during static balance tasks and the amount of spinal reflex excitability, over 86% of participants could be correctly classified as CAI patients. Similarly, Terada et al. [57] discovered that the combination of reduced spinal reflex excitability and self-reported disability could classify 72% of CAI participants. The authors of both aforementioned studies [57,58] as well as other authors [55,56] have consistently suggested that the consideration of spinal reflex excitability may be more important than consideration of other well-known classifying factors such as impaired joint kinesthesia or ankle joint laxity. In addition, altered spinal reflex excitability was associated with postural control deficits [31] and self-reported disability [29,30]: CAI patients with greater changes in reflex excitability also felt more disabled by their poor balance. All these observations point towards the fact that spinal reflex excitability plays an important role in CAI and constitutes a strong indicator for functional impairments in patients with CAI.

#### 4.1.2. Mechanisms of Reflex Inhibition in CAI Patients

The results of our meta-analysis indicate chronically reduced H-reflexes in CAI patients. At the spinal level, several mechanisms have been proposed to (task-specifically) adjust and modulate the size of the H-reflex such as presynaptic, reciprocal, recurrent, and Ib inhibition (for a review see [69]). Although the underlying mechanisms of reduced H-reflexes in CAI patients are not well understood, there is evidence for disturbed reflex propagation at both the pre- and postsynaptic level [32,53]. Sefton et al. [53] demonstrated that recurrent inhibition was enhanced in CAI patients, leading to a general depression of the α-motoneuron pool independent of whether subjects were standing in a single- or double-legged stance. Apart from these postsynaptic mechanisms, Sefton et al. [53] also found differences in presynaptic transmission at the spinal level between healthy controls and CAI-patients. When healthy controls switched from a double- to a single-legged stance, they reduced the amount of paired reflex depression (PRD). In contrast, CAI-patients did not modulate the amount of presynaptic inhibition when switching to the more demanding single-legged stance condition. 

In contrast to Sefton and colleagues [53], a recent study [32] proposed that CAI patients demonstrate disinhibition of spinal reflexes due to reduced presynaptic inhibition. At the same time, the authors did not find any differences in recurrent inhibition between participants with CAI and healthy controls. Although the study seems well-conducted it is difficult to interpret their results (i.e., facilitated H-reflexes in CAI) as they contradict most other studies in this field, as well as the outcome of the present meta-analysis demonstrating a reduction in spinal reflex excitability in patients with CAI. However, there is one important aspect in common: Thomson et al. [32] confirmed that CAI patients do not show task-specific modulation of presynaptic inhibition. Previously, it has been suggested that presynaptic inhibition provides an effective means for rapidly adapting to sudden environmental changes whereas postsynaptic inhibition may provide a more generalized and longer-lasting change in the spinal reflex circuitry [70]. In this sense, the postsynaptic downregulation of the motoneuron excitability by increased recurrent inhibition may contribute to the reduced H-reflexes seen in CAI-patients [53], probably irrespective of the postural condition in which the patients are measured. In contrast, the impaired modulation of presynaptic inhibition may only be apparent in paradigms in which H-reflexes were assessed in at least two different postural conditions. In doing so, Kim et al. [31,44] observed less modulation of the H-reflex (soleus and fibularis longus) in CAI patients than in healthy controls when switching from lying prone to a unipedal stance. The authors assumed that presynaptic mechanisms were altered in these CAI-patients. However, more importantly, it was demonstrated that the inability to modulate the H-reflex was significantly correlated with impaired balance control [31]. 

#### 4.1.3. Reduced Spinal Reflex Excitability as a Sign of “Arthrogenic Muscle Inhibition” in CAI Patients?

Reduced spinal reflex excitability in CAI patients, determined by lower amplitudes of H-reflexes, has been considered arthrogenic muscle inhibition (AMI), defined as an on-going reflexive inhibition of uninjured muscles surrounding the injured joint [71]. It was speculated that AMI is a protective mechanism of the central nervous system (CNS) to secure the injured ankle joint at the time of injury but does not seem to resolve completely as injury recovery proceeds [72]. As a result, prolonged inhibition of the dynamic ankle stabilizers may contribute to the chronic nature of poor neuromuscular control at the ankle joint in CAI patients. In this sense, AMI may be considered as an underlying neurophysiological mechanism of sensorimotor impairment following joint injury in general, and ankle joint injuries in particular [15,68,71]. Although many authors [27,28,34,38,39,44,52,55,56] may agree with this view because they consider reduced H-reflexes not only as an essential but as a sufficient criterion to classify (undoubtedly) AMI, this classification is at least questionable. It is well known that balance training reduces spinal reflex excitability too (for a review see [69]). Although most studies reported training-induced reductions in the H-reflex only when measured during postural task execution [73,74], there are also balance training studies that observed reduced spinal reflex excitability at rest [75]. Due to the fact that most chronic CAI patients have undergone balance exercises as part of their rehabilitation program, reduced H-reflexes as the only criterion might considerably bias this classification. Furthermore, it has to be considered that the inter-individual variability in the size of the H-reflex, and also the ratio of the H-reflex normalized to the maximal M-wave (Hmax/Mmax ratio), is extremely high [76] and further depends on the age of the subjects [77]. In addition, populations that display impaired motor control compared to healthy young adults such as elderly people, children born preterm, or persons with pathological conditions like Huntington’s disease, cerebral palsy or spasticity often demonstrate reduced inhibitory capacity at both the spinal [77,78,79,80] and cortical levels [81,82,83]. These examples illustrate that a reduction of spinal reflexes—as the data from the systematic analysis of CAI patients in the present study propose—should not be considered as a maladaptation per se and should not automatically be classified as AMI. In order to get a better idea about the functional consequences of reduced spinal reflex excitability in patients with CAI and to establish a better link between cause and effect, it is strongly recommended that future studies concentrate not only on reflex (and TMS) measurements at rest but also during the execution of postural (functional) tasks. Measurements that are performed at rest (or in a less-demanding control conditions) and during activity allow the modulatory range to be assessed by comparing adjustments during activity with the “baseline” level at rest. For both the spinal [80,84,85] and cortical levels [83,86] it was shown that aging as well as certain pathologies reduce the modulatory range, and thus the ability of the CNS to adapt to the task specific requirements. It may therefore be assumed that a chronic reflex inhibition such as AMI would result in a reduced capacity to task-specifically adjust and modulate the H-reflex. Recently, such a reduced H-reflex modulation was indeed shown in CAI patients [31]. Interestingly, the reduced ability to task-specifically modulate the H-reflex between lying prone and one-legged standing in CAI patients was associated with reduced stance stability (r = 0.578, *p* = 0.049). Furthermore, as mentioned above in Section 4.1.2, CAI-patients demonstrated an impaired capacity to modulate presynaptic inhibition between a single- and a double-legged stance [32,53]. These studies provide strong evidence for chronic malfunctioning of spinal reflex circuitries in CAI patients. 

### 4.2. Corticospinal Excitability Associated with CAI

There is no conclusive evidence that the supra-spinal excitability of ankle muscles in patients with CAI is altered relative to that of uninjured controls, as CMT measures across individual studies provide conflicting results. Pietrosimone and Gribble [35] first investigated the corticospinal excitability of the fibularis longus, and they reported higher CMT in individuals with CAI when compared with uninjured controls. Based on this observation, the authors [35] assumed that patients with CAI may encounter more difficulty in activating the fibularis longus muscle via corticospinal tract fibers, which may in turn lead to inadequate control of the ankle joint, resulting in ankle dysfunction and increased risk of recurrent ankle injuries. In addition, higher CMT was significantly associated with higher self-reported disability, indicating that the level of corticomotor excitability influences the patient’s perception of their ankle function [35]. In contrast, all subsequent studies [33,34,36,40] failed to confirm higher CMT’s in either the fibularis longus or soleus muscle in patients with CAI. However, three out of these four studies [33,34,36] nevertheless found alterations in different TMS-based parameters. McLeod et al. [34] observed lower amplitudes of the motor-evoked potential in the fibularis longus when measured during weak contractions. This supports the initial findings from Pietrosimone and Gribble [35] and suggests decreased ability to activate lower leg muscles via the corticospinal tract in patients with CAI. The reason for this reduced corticospinal connectivity might be related to observations from Kosik et al. [33], who discovered decreased corticomotor map area and volume of the fibularis longus muscle. In this study, a TMS mapping technique was used to estimate the size of the corticomotor representation, which was apparently smaller in CAI patients than in uninjured controls. Another mechanism, which might also contribute to reduced corticospinal connectivity in CAI patients, is the observation of Terada et al. [36] who reported greater cortical silent periods in the soleus muscle of CAI patients. The silent period is an indicator of GABAb-mediated intracortical inhibition and originates largely from activation of cortical inhibitory interneurons [87] although spinal mechanisms are also involved in the early part of the silent period [88]. Thus, the longer silent periods indicate increased intracortical inhibition, which may impede activation of lower leg muscles via the corticospinal tract in CAI patients. It is noted, however, that there is another TMS study [40] which failed to find alterations in the silent period of the fibularis longus. 

Collectively, these TMS studies suggest that supra-spinal adaptions may very well occur in patients with CAI and probably contribute to their sensorimotor impairments [33,34,35,36]. Supporting this, a recent magnetic resonance imaging study [89] assessed the white matter microstructure of the superior cerebellar peduncle by means of diffusion tensor imaging in patients with CAI and found lower white matter microstructure in CAI patients. At the same time, the CAI patients demonstrated worse postural control than healthy subjects. 

In summary, the limited number of TMS studies, the different test situations (measurements at rest versus measurements during voluntary muscle contractions while sitting or lying), and the rather non-specific muscle activations during testing may have prevented finding conclusive evidence, so far. In particular, the last point seems crucial: it is very well known that corticospinal excitability as well as inhibitory processes are task-specifically modulated [90,91,92]. It would, therefore, seem plausible that differences in supraspinal control between CAI patients and uninjured controls should be most obvious during tasks in which CAI patients reveal impaired motor control such as balance tasks. This may help to more clearly indicate the underlying neural mechanisms associated with CAI at the supraspinal level. 

### 4.3. Limitations 

Our findings indicate an overall impairment (reduction) of spinal reflexes with CAI, but there are inconsistencies when regarding supraspinal changes. Although our approach, analyzing solely CMT as one common TMS outcome parameter, is in line with the recommendation to avoid violation of the assumption of independence in the meta-analysis [51], the outcome or interpretation may change when considering other TMS measures. However, it has to be noted that each TMS measure of either soleus or fibularis longus, other than CMT, was only used in a single study, making it difficult to perform a meta-analysis. The findings of the present systematic review are limited to young adults with ages ranging from 19.8 to 26.5 years and thus, cannot be uncritically translated to other age groups. Furthermore, the present review did not incorporate non-English studies.

### 4.4. Recommendations for Future Research

The present systematic review of the literature found several points to be considered in future research investigating neural excitability in patients with CAI using TMS and/or H-reflex techniques. First, while studies included in the current investigation suggested that CAI may be associated with decreased spinal reflex excitability [27,28,44,52,55,56,57], the retrospective nature of these studies does not provide information on whether the reduced excitability is related to the development of CAI or is simply a consequence of CAI. Prospective studies are warranted to elucidate this link. In addition, it is not known how the neural changes occur following an initial ankle injury. It is recommended that this time course be established in order to make comparisons with knee-injury patients. In knee patients, reduction of quadriceps reflexes appears to be most severe in the acute stages of knee injury or surgery and these are slowly restored over time, although it is noted that the diminished level of reflexes may be still clinically significant in the long term (i.e., 18–33 months) [72]. Thus, a longitudinal study is needed to understand how neural excitability following initial ankle injury plays a role in developing CAI. Secondly, reporting outcome measures of spinal excitability should be better standardized to allow for between-study comparisons. A report of a recruitment curve may be preferred, from which multiple parameters of neural excitability can be extracted. Thirdly, studies investigating corticomotor excitability with TMS should be aware that the MEP is influenced by both spinal and supraspinal (cortical) excitability [93]. Thus, studies that want to assess cortical measures should combine TMS with other measures. Apart from this, it is strongly recommended that spinal as well as cortical changes accompanying CAI be assessed, not only at rest but also during activity (see text above for more details). Finally, a wide range of CAI populations should be examined in future studies. A majority of the studies included in the review recruited young adults in a university setting, which certainly limits the generalizability of the findings. This seems important considering the fact that CAI affects not only young but also older populations [1,4,94].

## 5. Conclusions

The current systematic review with meta-analysis is the first to demonstrate moderate-quality evidence that patients with CAI presented decreased spinal reflex excitability in the soleus and fibularis longus, as determined with Hmax/Mmax ratios. The diminished spinal reflexes may contribute to the occurrence of AMI. However, limited data about reflex modulation and task-specific adjustments of spinal reflexes makes it impossible to clarify this question at this stage, as reflex inhibition is not maladaptive per se (for details see the text above). On the other hand, there is limited evidence to determine whether supraspinal excitability is altered in CAI patients when considering only CMT. However, when taking into account a more global picture of all the supraspinal changes that were detected by means of TMS (and diffusion tensor imaging), it seems reasonable to assume that descending drive from the motor cortex to ankle muscles is reduced in patients with CAI. 

## Figures and Tables

**Table 1:** Characteristics of included studies.

Author (Year)	Study Design	Inclusion Criteria		Participant Characteristics		Stimulation Settings	Outcome
		CAI Group	Comparison Group	CAI Group	Comparison Group		
McVey (2005)	Case- control	≥5 “yes” responses on AII	Uninjured: No history of ankle injury or significant lower extremity injury or surgery	15 unilateral CAI patients (8 females, 26.5 ± 126.5 years, 173 ± 6.8 cm, 70 ± 7.2 kg)	14 uninjured (13 females, 21.3 ± 2.5 years,166 ± 5.4 cm, 61 ± 6.4 kg)	Unipolar stimulating electrode to stimulate the sciatic nerve with 1 ms squared wave pulse that were 10 s apart by increasing stimulus intensity in 0.2 V until H_max_ and M_max_ were obtained	H_max_:M_max_ ratios of soleus, fibularis longus, and tibialis anterior measured in a prone position
Sefton (2008)	Case- control	>1 ankle sprain in the previous year, recurring symptoms, and difficulty in >1 area in the FADI or 2 areas in the FADI-Sport	Uninjured: No history of ankle injury and no incidence of acute or chronic lower extremity injuries	22 CAI patients (17 females, 22.3 years, 167.6 cm, 69.8 kg) *Measures of standard deviation were not reported*	21 uninjured (16 females, 21.9 years, 166.0 cm, 64.1 kg) *Measures of standard deviation were not reported*	Unipolar stimulating electrode placed over the popliteal fossa to stimulate the posterior tibial nerve with 1 ms squared wave pulse that were 10 to 20 s apart by slowly increasing stimulus intensity	H_max_:M_max_ ratio of soleus during bipedal stance and percent changes in paired reflex depression and recurrent inhibition of soleus measured both in unipedal and bipedal stances
Doeringer (2009)	Case-control with crossover	A history of ankle sprain, episodes of “giving way” and feelings of instability (≥3 “yes” responses on AII)	Uninjured: No history of ankle sprain or other lower extremity injuries to the tested limb	12 CAI patients (9 females, 23 ± 1 years, 168.7 ± 9.8 cm, 73.4 ± 20.0 kg)	12 uninjured (7 females, 23 ± 1 years, 171.7 ± 7.0 cm, 77.9 ± 14.9 kg)	Bipolar (bar) stimulating electrode placed over the popliteal fossa to stimulate the posterior tibial nerve with 1 ms squared wave pulses that were 20 s apart by increasing stimulus intensity in 0.2 to 0.5 V increments until H_max_ and M_max_ were obtained	H_max_:M_max_ ratio of soleus measured in a reclining position with 120° of hip flexion and 60° of knee flexion, and the neutral position of the ankle
Palmieri-Smith (2009)	Case-control	All CAI subjects that were physically active (Tegner score of 5 or 6) met the criteria set forth by both Functional Ankle Instability Questionnaire and AII.	Uninjured: All control subjects were physically active (Tegner score of 5 or 6), but other criteria were not specified.	21 unilateral CAI patients (18 females, 21 ± 2 years, 171 ± 7 cm, 65 ± 9 kg)	21 uninjured (18 females, 21 ± 3 years, 169 ± 9 cm, 64 ± 10 kg)	Unipolar stimulating electrode placed over the popliteal fossa to stimulate the sciatic nerve with 1 ms squared wave pulses that were 10 s apart by increasing stimulus intensity in 0.2 V increments until H_max_ and M_max_ were obtained	H_max_:M_max_ ratio of fibularis longus measured in prone position
Doeringer (2010)	Case-control with crossover	A history of ankle sprain, episodes of “giving way” and feelings of instability (≥3 “yes” responses on AII)	Uninjured: No history of ankle sprain or other lower extremity injuries to the tested limb	12 CAI patients (9 females, 23 ± 1 years, 168.7 ± 9.8 cm, 73.4 ± 20.0 kg)	12 uninjured (7 females, 23 ± 1 years, 171.7 ± 7.0 cm, 77.9 ± 14.9 kg)	Bipolar (bar) stimulating electrode placed over the popliteal fossa to stimulate the sciatic nerve with 1 ms squared wave pulses that were 20 s apart by increasing stimulus intensity in 0.2 to 0.5 V increments until H_max_ and M_max_ were obtained	H_max_:M_max_ ratio of fibularis longus and tibialis anterior measured in a reclining position with 120° of hip flexion, 60° of knee flexion, and the neutral position of the ankle
Sefton (2011)	Case- control with repeated measures	>1 ankle sprain in the previous year, recurring symptoms, and difficulty in >1 area in the FADI or 2 areas in the FADI-Sport	Uninjured: No history of ankle injury and no incidence of acute or chronic lower extremity injuries	12 CAI patients (8 females, 21.2 ± 2.1 years, 165.1 ± 8.9 cm, 67.2 ± 9.4 kg)	9 uninjured (6 females, 20.8 ± 1.3 years, 167.3 ± 7.9 cm, 62.8 ± 10.3 kg)	Unipolar stimulating electrode placed over the popliteal fossa to stimulate the posterior tibial nerve with 1 ms squared wave pulse that were 10 to 20 s apart by slowly increasing stimulus intensity	H_max_:M_max_ ratio of soleus during bipedal stance and percent changes in paired reflex depression and recurrent inhibition of soleus measured both in unipedal and bipedal stances
Kim (2012)	Case-control	A history of at least 1 lateral ankle sprain (1-yr old or greater), episodes of “giving way”, feelings of instability (≥4 “yes” responses on AII), and self-reported ankle disability (≤90% on FAAM and ≤80% on the FAAM-Sport)	Uninjured: No history of ankle injury or significant lower extremity injury or surgery, and any limitation of ankle function	16 unilateral CAI patients (6 females, 21.0 ± 6.9 years, 173.9 ± 7.4 cm, 72.6 ± 11.9 kg)	15 uninjured (6 females 19.9 ± 4.3 years, 175.8 ± 9.7 cm, 71.3 ± 17.8 kg)	Unipolar stimulating electrode placed over the superior popliteal fossa to stimulate the sciatic nerve with 1 ms squared wave pulses that were at least 12 s apart by increasing stimulus intensity in 0.2 V increments until H_max_ was obtained, then 1.0 V increments until M_max_ plateaued	H_max_:M_max_ ratio of soleus and fibularis longus measured in 3 body positions: prone, bipedal, and unipedal stances
Pietro- simone (2012)	Case-control	A history of at least 2 unilateral ankle sprains and self-reported function (<90% on FADI, <80% on FADI-Sport)	Uninjured: No ankle instability and self-reported functions (>95% on FADI, >85% on FADI-Sport)	10 unilateral CAI patients (6 females, 21.2 ± 1.2 years, 175.1 ± 9.7 cm, 77.1 ± 13.6 kg)	10 uninjured (6 females 21.2 ± 2.3 years, 172.3 ± 8.9 cm, 73.4 ± 7.2 kg)	Double-cone coil placed over the contralateral vertex of the cranium relative to the involved limb to deliver a single magnetic pulse of a maximum magnetic stimulus of 1.4 Tesla that were 15 s apart between trials	Resting motor threshold expressed as a percentage of 2 Tesla of fibularis longus measured in the seated position with 85° of hip flexion, 10° of knee flexion, and 10° of ankle plantar flexion
Needle (2013)	Case- control	A history of at least 1 unilateral ankle sprain (≤25 on CAIT)	Uninjured: No history of ankle injury and a score of >27 on CAIT)	12 unilateral CAI patients (6 females, 20.9 ± 4.1 years, 170.6 ± 10.1 cm, 72.5 ± 15.0 kg)	12 uninjured (6 females 21.2 ± 2.6 years, 172.7 ± 8.5 cm, 70.0 ± 15.0 kg)	A figure-8 coil placed over the contralateral vertex of the cranium relative to the involved limb to deliver a single magnetic pulse of a maximum magnetic stimulus of 1.4 Tesla that were 5 s apart between trials	AMT of soleus, fibularis longus, and tibialis anterior measured in the seated position with pronation of ankle at 15% of maximal effort of fibularis longus activity
Kim (2015)	Case-control with crossover	A history of at least 1 lateral ankle sprain (1-yr old or greater), episodes of “giving way”, feelings of instability (≥4 “yes” responses on AII), and self-reported ankle disability (≤90% on FAAM and ≤80% on the FAAM-Sport)	Uninjured: No history of ankle injury or lower extremity injury or surgery and any limitation of ankle function	15 CAI patients (6 females, 22.6 ± 5.8 years, 174.7 ± 8.1 cm, 74.9 ± 12.8 kg)	15 uninjured (6 females, 23.8 ± 5.8 years, 171.9 ± 9.9 cm, 68.9 ± 15.5 kg)	Unipolar stimulating electrode placed over the superior popliteal fossa to stimulate the sciatic nerve with 1 ms squared wave pulses that were at least 12 s apart by increasing stimulus intensity in 0.2 V increments until H_max_ was obtained, then 1.0 V increments until M_max_ plateaued	H_max_:M_max_ ratio of soleus and fibularis longus measured in 3 body positions: prone, bipedal and unipedal stances
McLeod (2015)	Case-control	A history of at least 1 acute lateral ankle sprain, resulting in swelling, pain, and/or temporary loss of function but not within the 3 months) and >2 episodes of the ankle “giving way” in the 6 months (≤80% on the FAAM-Sport)	Uninjured: No history of ankle injury and a score of 100% on the FAAM-Sport	21 CAI patients (12 females, 20.8 ± 1.6 years, 171.6 ± 11.4 cm, 68.8 ± 11.9 kg)	24 uninjured (17 females, 22.5 ± 2.9 years, 172.4 ± 10.9 cm, 69.2 ± 12.3 kg)	For H-reflex testing, the unipolar stimulating electrode to stimulate (1) the sciatic nerve for fibularis longus and (2) the femoral nerve for vastus medialis separately by increasing stimulus intensity in 0.2 V increments until H_max_ was obtained For TMS testing, the double-cone coil placed over the contralateral vertex of the cranium relative to the involved limb to deliver a brief magnetic stimulus of a maximum magnetic stimulus of 1.4 Tesla	H_max_:M_max_ ratios of both fibularis longus and vastus medialis measured in a supine position For the fibularis longus AMT and 5 MEP responses at varying levels of intensity, measured in the seated position with 85° of hip flexion, 10° of knee flexion, and 10° of ankle plantar flexion For the vastus medialis, AMT and 5 MEP responses at varying levels of intensity, measured in the seated position with 85° of hip flexion and 90° of knee flexion
Bowker (2016)	Case-control	A history of at least 1 ankle sprain, resulting in swelling, pain, and/or temporary loss of function), ≥2 episodes of the ankle “giving way” within the 6 months, perceived ankle instability, and dysfunction during daily living activities (≥5 “yes” responses on AII and scores of ≥11 on IdFAI)	Uninjured: No history of ankle sprain and a score of 0 on both AII and IdFAI Copers: History of ankle sprains, but no reported episode of the ankle “giving way”, perceived instability, or loss of function without modifying physical activity, <5 “yes” responses on AII, and scores of <11 on IdFAI	37 CAI patients (19 females, 22 ± 3.5 years, 25.2 ± 3.8 body mass index)	26 uninjured (17 females, 21.6 ± 3.2 years, 23.7 ± 2.8 body mass index 30 copers (17 females, 21.9 ± 4.3 years, 26.2 ± 6.3 body mass index)	Unipolar stimulating electrode placed over the proximal lateral popliteal fossa to stimulate the posterior tibial nerve with 1 ms squared wave pulse that were 10 s apart by increasing or decreasing the stimulus intensity in 0.2 V increments until H_max_ was obtained, then 1.0 V increments until M_max_ plateaued	H_max_:M_max_ ratio of soleus measured in the seated position with 90° of hip flexion, 90° of knee flexion, 90° of ankle plantar flexion
Terada (2016)	Case-control	A history of at least 2 significant ankle sprains, resulting in swelling, pain, and/or temporary loss of function, ≥2 episodes of the ankle “giving way” within the 6 months, perceived ankle instability, and dysfunction during daily living activities (≥4 “yes” responses on AII and scores of ≥11 on IdFAI)	Uninjured: No history of ankle sprain and a score of 0 on both the AII and IdFAI	16 CAI patients (6 females, 22.2 ± 3.6 years, 25.8 ± 2.5 body mass index)	17 uninjured (9 females, 21.2 ± 3.0 years, 24.7 ± 3.1 body mass index)	For M_max_ of the fibularis longus, the unipolar stimulating electrode placed over the proximal lateral popliteal fossa to stimulate the posterior tibial nerve with 1 ms squared wave pulse by increasing the stimulus intensity in 1.0 V increments until M_max_ plateaued For TMS testing, the double-cone coil placed over the contralateral vertex of the cranium relative to the involved limb to deliver a series of magnetic stimuli of 1.0 Tesla	AMT, MEP_120%_:M_max_ ratio, and CSP: MEP_120%_ ratio of soleus measured in the seated position with 90° of knee flexion, 90° of ankle dorsiflexion
Kosik (2017)	Case-control	A history of at least 1 acute lateral ankle sprain, resulting in swelling, pain, and/or temporary loss of function and ≥2 episodes of the ankle “giving way” within the 6 months (≥5 “yes” responses on AII and scores of ≥11 on IdFAI ≤24 on CAIT)	Uninjured: No history of lower extremity injuries and a score 0 on both the AII and IdFAI, and 30 on CAIT	18 CAI patients (14 females, 23.8 ± 3.6 years, 169.6 ± 7.5 cm, 73.1 ± 12.0 kg)	16 uninjured (10 females, 21.1 ± 2.2 years, 168.6 ± 13.4 cm, 66.5 ± 10.2 kg)	For H-reflex testing, the unipolar stimulating electrode to stimulate the proximal common fibular nerve with 1 ms squared wave pulse by increasing or decreasing the stimulus intensity in 0.2 V increments until H_max_ was obtained, then 1.0 V increments until M_max_ plateaued For TMS testing, The double-cone coil placed over the contralateral vertex of the cranium relative to the involved limb to deliver a series of magnetic stimuli of 1 Tesla that were 10 s apart	H_max_:M_max_ ratio of fibularis longus measured in the prone position MEP_100%_:M_max_ ratio and corticomotor map outcomes for fibularis longus: (1) size of corticomotor map area, (2) volume of corticomotor map, (3) location of cortical representation, measured in the seated position with 75° of hip flexion, 60° of knee flexion, 80° of ankle plantar flexion
Terada (2017)	Case- control	CAI subgroups: (1) PI-RAS: A history of at least 2 previous ankle sprains and ≥2 episodes of the ankle “giving way” within the 6 months (≥5 “yes” responses on AII and scores of ≥11 on IdFAI) (2) PI: A history of one previous ankle sprain and ≥2 episodes of the ankle “giving way” within the 6 months (≥5 “yes” responses on AII and scores of ≥11 on IdFAI) (3) RAS: A history of at least 2 previous ankle sprains, but no episode of the ankle “giving way”, <5 “yes” responses on AII, and scores of <11 on IdFAI	Uninjured: No history of ankle sprain and a score 0 on both the AII and IdFAI Copers: A history of one previous ankle sprain, report of returning to full activity for at least 12 months following an initial ankle sprain without recurrent injury, no reported episode of the ankle “giving way”, perceived instability, or loss of function without modifying physical activity, <5 “yes” responses on AII, and scores of <11 on IdFAI	25 PI-RAS patients (11 females, 22.5 ± 4.0 years, 171.4 ± 8.7 cm, 76.2 ± 14.8 kg, 25.8 ± 3.6 body mass index) 13 PI patients (9 females, 20.8 ± 1.6 years, 165.8 ± 6.5 cm, 65.7 ± 11.8 kg, 23.9 ± 3.8 body mass index) 12 RAS patients (6 females, 22.2 ± 4.8 years, 171.0 ± 10.3 cm, 79.1 ± 7.8 kg, 26.7 ± 7.6 body mass index)	26 uninjured (17 females, 21.6 ± 3.2 years, 166.1 ± 8.1 cm, 66.2 ± 13.1 kg, 23.8 ± 3.0 body mass index) 18 copers (11 females, 21.6 ± 4.0 years, 169.6 ± 11.3 cm, 72.4 ± 17.3 kg, 24.9 ± 3.8 body mass index)	For H-reflex testing, the unipolar stimulating electrode to stimulate the posterior tibial nerve with 1 ms squared wave pulse that were 10 s apart by increasing the stimulus intensity in 0.2 V increments until H_max_ was obtained, then 1.0 V increments until M_max_ plateau	H_max_:M_max_ ratio of soleus measured in the seated position with 90° of hip flexion, 90° of knee flexion, and 90° of ankle plantar flexion
Otzel (2019)	Case-control with crossover	A history of at least 1 moderate ankle sprain requiring immobilization, no formal rehabilitation, at least one recurrent ankle sprain 3-6 months prior to participation, perceived pain, ankle instability or weakness, and self-reported functional limitations (≤90% on FADI and ≤80% on the FADI-Sport)	Uninjured age-matched control *Specific inclusion criteria not reported*	10 CAI patients (6 females, 20.7 ± 1.3 years, 169.4 ± 10.7 cm, 66.0 ± 10.1 kg)	10 uninjured (7 females, 19.8 ± 0.7 years, 165.6 ± 9.2 cm, 59.1 ± 10.7 kg)	For H-reflex testing, the unipolar stimulating electrode to stimulate the posterior tibial nerve with 1 ms squared wave pulse that were 10 s apart by increasing the stimulus intensity in 0.2 V increments until H_max_ was obtained, then continued until M_max_ plateau	H_max_:M_max_ ratio of soleus measured in the seated position with 30° of hip flexion, 90° of knee flexion, 90° of ankle plantar flexion
Thompson (2019)	Case-control	A history of at least 1 significant ankle sprain, causing inflammatory symptoms and disrupted activity), the most recent ankle sprain occurred less than 3 months prior to study participation, reports of episodes of the ankle “giving way” and/or recurrent pain and/or perceived ankle instability, and dysfunction during daily living activities (≥5 “yes” responses on AII and scores <24 on CAIT)	Uninjured: No history of an ankle sprain Copers: History of ankle sprains, but no report of recurrent injuries, episode of the ankle “giving way”, and/or perceived instability	12 CAI patients (4 females, 25.2 ± 3.7 years, 177.7 ± 8.1 cm, 75.8 ± 14.8 kg)	12 uninjured (4 females, 23.3 ± 4.5 years, 171.6 ± 6.2 cm, 74.3 ± 10.2 kg) 12 copers (4 females, 24.2 ± 4.7 years, 172.7 ± 8.2 cm, 71.4 ± 6.9 kg)	For H-reflex testing, the unipolar stimulating electrode to stimulate the posterior tibial nerve with 1 ms squared wave pulse that was 10–15 s apart	Soleus H_max_:M_max_ ratio and slope of recruitment curve during bipedal stance Soleus H_50%_:M_max_ ratio and percent changes in presynaptic inhibition and recurrent inhibition, measured both in unipedal and bipedal stances

Abbreviations: CAI, chronic ankle instability; AII, Ankle Instability Instrument; Hmax:Mmax ratio, maximal Hoffmann reflex and maximal muscle response ratio; FAAM, Foot and Ankle Ability Measure; FADI, Foot and Ankle Disability Index; TMS, transcranial magnetic stimulation; AMT, active motor threshold; MEP, motor evoked potential; IdFAI, Identification of Functional Ankle Instability instrument; CSP, cortical silent period; CAIT, Cumberland Ankle Instability Tool; PI-RAS, perceived instability in combination with recurrent ankle sprain; PI, perceived instability; RAS, recurrent ankle sprain.

**Table 2:** Quality assessment of included studies.

Study		Reporting							External Validity		Internal Validity Bias		Internal Validity Confounding		
	Quality Index Score (%)	1. Hypothesis Clearly Described?	2. Main Outcomes Clearly Described?	3. Characteristics of the Patients included Clearly Described?	5. Distribution of Principle Confounder of Each Group Clearly Described?	6. Main Findings Clearly Described?	7. Estimates of Random Variability Provided for the Main Outcomes?	10. Actual Probability Values Reported for Main Outcomes?	11. Were the Subjects Asked to Participate Representative of the Entire Population?	12. Were the Subjects who Were Prepared to Participate Representative of the Entire Population?	16. Was it Clear if the Results Were Based on “Data Dredging’?	18. Were the Statistical Tests Appropriate?	20. Were the Main Outcome Measures Valid and Reliable?	21. Were all Patients and Controls Recruited from the Same Population?	22. Were all Patients and Controls Recruited over the Same Time Period?
McVey (2005)	60.0	+	+	-	+	+	+	+	-	-	+	+	+	-	-
Sefton (2008)	66.7	-	+	-	++	+	+	+	-	-	+	+	+	+	-
Doeringer (2009)	66.7	+	+	-	+	+	+	+	-	-	+	+	+	+	-
Palmieri-Smith (2009)	66.7	+	+	-	++	+	+	+	-	-	+	+	+	-	-
Doeringer (2010)	66.7	+	+	-	+	+	+	+	-	-	+	+	+	+	-
Sefton (2011)	60.0	+	+	-	+	+	+	+	-	-	+	+	+	-	-
Kim (2012)	73.3	+	+	+	++	+	+	+	-	-	+	+	+	-	-
Pietro- simone (2012)	66.7	+	+	-	++	+	+	+	-	-	+	+	+	-	-
Needle (2013)	46.7	-	-	-	++	-	+	+	-	-	+	+	-	+	-
Kim (2015)	73.3	+	+	+	++	+	+	+	-	-	+	+	+	-	-
McLeod (2015)	60.0	+	+	-	+	+	+	+	-	-	+	+	+	-	-
Bowker (2016)	73.3	+	+	+	+	+	+	+	-	-	+	+	+	+	-
Terada (2016)	73.3	+	+	+	+	+	+	+	-	-	+	+	+	+	-
Kosik (2017)	73.3	+	+	+	+	+	+	+	-	-	+	+	+	+	-
Terada (2017)	73.3	+	+	+	+	+	+	+	-	-	+	+	+	+	-
Otzel (2019)	60.0	+	+	+	+	+	+	+	-	-	+	+	-	-	-
Thompson (2019)	60.0	+	+	+	+	+	+	+	-	-	+	+	-	-	-
Average (SD)	65.9 (7.4)														

A zero score, as reflected by the negative sign (-) in the table, was given to an item that was not satisfied, while the items that were satisfied scored one point, as reflected by the positive sign (+); two points could be earned for item 5 as reflected by the double positive sign (++).

## References

[1] Gribble P.A., Bleakley C.M., Caulfield B.M., Docherty C.L., Fourchet F., Fong D.T., Hertel J., Hiller C.E., Kaminski T.W., McKeon P.O. (2016). Evidence review for the 2016 International Ankle Consortium consensus statement on the prevalence, impact and long-term consequences of lateral ankle sprains. Br. J. Sports Med..

[2] Gribble P.A., Delahunt E., Bleakley C.M., Caulfield B., Docherty C.L., Fong D.T., Fourchet F., Hertel J., Hiller C.E., Kaminski T.W. (2014). Selection criteria for patients with chronic ankle instability in controlled research: A position statement of the International Ankle Consortium. J. Athl. Train..

[3] Attenborough A.S., Sinclair P.J., Sharp T., Greene A., Stuelcken M., Smith R.M., Hiller C.E. (2016). A snapshot of chronic ankle instability in a cohort of netball players. J. Sci. Med. Sport.

[4] Tanen L., Docherty C.L., Van Der Pol B., Simon J., Schrader J. (2014). Prevalence of chronic ankle instability in high school and division I athletes. Foot Ankle Spec..

[5] Attenborough A.S., Hiller C.E., Smith R.M., Stuelcken M., Greene A., Sinclair P.J. (2014). Chronic ankle instability in sporting populations. Sports Med..

[6] Simon J., Hall E., Docherty C. (2014). Prevalence of chronic ankle instability and associated symptoms in university dance majors: An exploratory study. J. Dance Med. Sci..

[7] Hubbard-Turner T., Turner M.J. (2015). Physical Activity Levels in College Students with Chronic Ankle Instability. J. Athl. Train..

[8] Hubbard-Turner T., Wikstrom E.A., Guderian S., Turner M.J. (2015). An Acute Lateral Ankle Sprain Significantly Decreases Physical Activity across the Lifespan. J. Sports Sci. Med..

[9] Houston M.N., Hoch J.M., Hoch M.C. (2015). Patient-Reported Outcome Measures in Individuals with Chronic Ankle Instability: A Systematic Review. J. Athl. Train..

[10] Lee M., Kwon J.W., Choi W.J., Lee J.W. (2015). Comparison of Outcomes for Osteochondral Lesions of the Talus with and Without Chronic Lateral Ankle Instability. Foot Ankle Int..

[11] Bischof J.E., Spritzer C.E., Caputo A.M., Easley M.E., DeOrio J.K., Nunley J.A., DeFrate L.E. (2010). In vivo cartilage contact strains in patients with lateral ankle instability. J. Biomech..

[12] Valderrabano V., Horisberger M., Russell I., Dougall H., Hintermann B. (2009). Etiology of ankle osteoarthritis. Clin. Orthop. Relat. Res..

[13] Valderrabano V., Hintermann B., Horisberger M., Fung T.S. (2006). Ligamentous posttraumatic ankle osteoarthritis. Am. J. Sports Med..

[14] Hertel J. (2002). Functional Anatomy, Pathomechanics, and Pathophysiology of Lateral Ankle Instability. J. Athl. Train..

[15] Hertel J. (2008). Sensorimotor deficits with ankle sprains and chronic ankle instability. Clin. Sports Med..

[16] Aman J.E., Elangovan N., Yeh I.L., Konczak J. (2014). The effectiveness of proprioceptive training for improving motor function: A systematic review. Front. Hum. Neurosci..

[17] Schiftan G.S., Ross L.A., Hahne A.J. (2015). The effectiveness of proprioceptive training in preventing ankle sprains in sporting populations: A systematic review and meta-analysis. J. Sci. Med. Sport.

[18] Rios J.L., Gorges A.L., dos Santos M.J. (2015). Individuals with chronic ankle instability compensate for their ankle deficits using proximal musculature to maintain reduced postural sway while kicking a ball. Hum. Mov. Sci..

[19] Terada M., Ball L.M., Pietrosimone B.G., Gribble P.A. (2016). Altered visual focus on sensorimotor control in people with chronic ankle instability. J. Sports Sci..

[20] Terada M., Bowker S., Thomas A.C., Pietrosimone B., Hiller C.E., Rice M.S., Gribble P.A. (2015). Alterations in stride-to-stride variability during walking in individuals with chronic ankle instability. Hum. Mov. Sci..

[21] Yen S.C., Corkery M.B., Donohoe A., Grogan M., Wu Y.N. (2016). Feedback and Feedforward Control During Walking in Individuals with Chronic Ankle Instability. J. Orthop. Sports Phys. Ther..

[22] Hertel J., Olmsted-Kramer L.C. (2007). Deficits in time-to-boundary measures of postural control with chronic ankle instability. Gait Posture.

[23] Delahunt E., Monaghan K., Caulfield B. (2006). Altered neuromuscular control and ankle joint kinematics during walking in subjects with functional instability of the ankle joint. Am. J. Sports Med..

[24] Doherty C., Bleakley C., Hertel J., Caulfield B., Ryan J., Sweeney K., Patterson M.R., Delahunt E. (2015). Lower Limb Interjoint Postural Coordination One Year after First-Time Lateral Ankle Sprain. Med. Sci. Sports Exerc..

[25] Hass C.J., Bishop M.D., Doidge D., Wikstrom E.A. (2010). Chronic ankle instability alters central organization of movement. Am. J. Sports Med..

[26] Terada M., Pietrosimone B.G., Gribble P.A. (2014). Alterations in neuromuscular control at the knee in individuals with chronic ankle instability. J. Athl. Train..

[27] McVey E.D., Palmieri R.M., Docherty C.L., Zinder S.M., Ingersoll C.D. (2005). Arthrogenic muscle inhibition in the leg muscles of subjects exhibiting functional ankle instability. Foot Ankle Int..

[28] Palmieri-Smith R.M., Hopkins J.T., Brown T.N. (2009). Peroneal activation deficits in persons with functional ankle instability. Am. J. Sports Med..

[29] Harkey M., McLeod M.M., Terada M., Gribble P.A., Pietrosimone B.G. (2016). Quadratic Association between Corticomotor and Spinal-Reflexive Excitability and Self-Reported Disability in Participants with Chronic Ankle Instability. J. Sport Rehabil..

[30] Kim K.M., Hart J.M., Saliba S.A., Hertel J. (2016). Relationships between self-reported ankle function and modulation of Hoffmann reflex in patients with chronic ankle instability. Phys. Ther. Sport.

[31] Kim K.M., Hart J.M., Saliba S.A., Hertel J. (2016). Modulation of the Fibularis Longus Hoffmann Reflex and Postural Instability Associated with Chronic Ankle Instability. J. Athl. Train..

[32] Thompson C.S., Hiller C.E., Schabrun S.M. (2019). Altered spinal-level sensorimotor control related to pain and perceived instability in people with chronic ankle instability. J. Sci. Med. Sport.

[33] Kosik K.B., Terada M., Drinkard C.P., McCann R.S., Gribble P.A. (2017). Potential Corticomotor Plasticity in Those with and without Chronic Ankle Instability. Med. Sci. Sports Exerc..

[34] McLeod M.M., Gribble P.A., Pietrosimone B.G. (2015). Chronic Ankle Instability and Neural Excitability of the Lower Extremity. J. Athl. Train..

[35] Pietrosimone B.G., Gribble P.A. (2012). Chronic ankle instability and corticomotor excitability of the fibularis longus muscle. J. Athl. Train..

[36] Terada M., Bowker S., Thomas A.C., Pietrosimone B., Hiller C.E., Gribble P.A. (2016). Corticospinal Excitability and Inhibition of the Soleus in Individuals with Chronic Ankle Instability. PM R.

[37] Needle A.R., Lepley A.S., Grooms D.R. (2017). Central Nervous System Adaptation after Ligamentous Injury: A Summary of Theories, Evidence, and Clinical Interpretation. Sports Med..

[38] Doeringer J.R., Hoch M.C., Krause B.A. (2009). The Effect of Focal Ankle Cooling on Spinal Reflex Activity in Individuals with Chronic Ankle Instability. Athl. Train. Sports Health Care.

[39] Doeringer J.R., Hoch M.C., Krause B.A. (2010). Ice application effects on peroneus longus and tibialis anterior motoneuron excitability in subjects with functional ankle instability. Int. J. Neurosci..

[40] Needle A.R., Palmer J.A., Kesar T.M., Binder-Macleod S.A., Swanik C.B. (2013). Brain regulation of muscle tone in healthy and functionally unstable ankles. J. Sport Rehabil..

[41] Moher D., Liberati A., Tetzlaff J., Altman D.G., Group P. (2009). Preferred reporting items for systematic reviews and meta-analyses: The PRISMA statement. PLoS Med..

[42] Palmieri R.M., Ingersoll C.D., Hoffman M.A. (2004). The hoffmann reflex: Methodologic considerations and applications for use in sports medicine and athletic training research. J. Athl. Train..

[43] Rossini P.M., Burke D., Chen R., Cohen L.G., Daskalakis Z., Di Iorio R., Di Lazzaro V., Ferreri F., Fitzgerald P.B., George M.S. (2015). Non-invasive electrical and magnetic stimulation of the brain, spinal cord, roots and peripheral nerves: Basic principles and procedures for routine clinical and research application. An updated report from an I.F.C.N. Committee. Clin. Neurophysiol..

[44] Kim K.M., Ingersoll C.D., Hertel J. (2012). Altered postural modulation of Hoffmann reflex in the soleus and fibularis longus associated with chronic ankle instability. J. Electromyogr. Kinesiol..

[45] Downs S.H., Black N. (1998). The feasibility of creating a checklist for the assessment of the methodological quality both of randomised and non-randomised studies of health care interventions. J. Epidemiol. Community Health.

[46] Simpson J.D., Stewart E.M., Macias D.M., Chander H., Knight A.C. (2018). Individuals with chronic ankle instability exhibit dynamic postural stability deficits and altered unilateral landing biomechanics: A systematic review. Phys. Ther. Sport.

[47] Moisan G., Descarreaux M., Cantin V. (2017). Effects of chronic ankle instability on kinetics, kinematics and muscle activity during walking and running: A systematic review. Gait Posture.

[48] McKeon J.M., McKeon P.O. (2012). Evaluation of joint position recognition measurement variables associated with chronic ankle instability: A meta-analysis. J. Athl. Train..

[49] Munn J., Sullivan S.J., Schneiders A.G. (2010). Evidence of sensorimotor deficits in functional ankle instability: A systematic review with meta-analysis. J. Sci. Med. Sport.

[50] Cohen J. (2013). Statistical Power Analysis for the Behavioral Sciences.

[51] Borenstein M., Hedges L.V., Higgins J.P.T., Rothstein H.R. (2009). Introduction to Meta-Analysis.

[52] Kim K.M., Ingersoll C.D., Hertel J. (2015). Facilitation of Hoffmann reflexes of ankle muscles in prone but not standing positions by focal ankle-joint cooling. J. Sport Rehabil..

[53] Sefton J.M., Hicks-Little C.A., Hubbard T.J., Clemens M.G., Yengo C.M., Koceja D.M., Cordova M.L. (2008). Segmental spinal reflex adaptations associated with chronic ankle instability. Arch. Phys. Med. Rehabil..

[54] Sefton J.M., Yarar C., Hicks-Little C.A., Berry J.W., Cordova M.L. (2011). Six weeks of balance training improves sensorimotor function in individuals with chronic ankle instability. J. Orthop. Sports Phys. Ther..

[55] Bowker S., Terada M., Thomas A.C., Pietrosimone B.G., Hiller C.E., Gribble P.A. (2016). Neural Excitability and Joint Laxity in Chronic Ankle Instability, Coper, and Control Groups. J. Athl. Train..

[56] Otzel D.M., Hass C.J., Wikstrom E.A., Bishop M.D., Borsa P.A., Tillman M.D. (2019). Motoneuron Function Does Not Change Following Whole-Body Vibration in Individuals with Chronic Ankle Instability. J. Sport Rehabil..

[57] Terada M., Bowker S., Hiller C.E., Thomas A.C., Pietrosimone B., Gribble P.A. (2017). Quantifying levels of function between different subgroups of chronic ankle instability. Scand. J. Med. Sci. Sports.

[58] Sefton J.M., Hicks-Little C.A., Hubbard T.J., Clemens M.G., Yengo C.M., Koceja D.M., Cordova M.L. (2009). Sensorimotor function as a predictor of chronic ankle instability. Clin. Biomech..

[59] Diekfuss J.A., Grooms D.R., Yuan W., Dudley J., Barber Foss K.D., Thomas S., Ellis J.D., Schneider D.K., Leach J., Bonnette S. (2019). Does brain functional connectivity contribute to musculoskeletal injury? A preliminary prospective analysis of a neural biomarker of ACL injury risk. J. Sci. Med. Sport.

[60] Pelletier R., Bourbonnais D., Higgins J. (2018). Nociception, pain, neuroplasticity and the practice of Osteopathic Manipulative Medicine. Int. J. Osteopath. Med..

[61] Rio E., Kidgell D., Moseley G.L., Gaida J., Docking S., Purdam C., Cook J. (2016). Tendon neuroplastic training: Changing the way we think about tendon rehabilitation: A narrative review. Br. J. Sports Med..

[62] Grooms D., Appelbaum G., Onate J. (2015). Neuroplasticity following anterior cruciate ligament injury: A framework for visual-motor training approaches in rehabilitation. J. Orthop. Sports Phys. Ther..

[63] Gokeler A., Neuhaus D., Benjaminse A., Grooms D.R., Baumeister J. (2019). Principles of Motor Learning to Support Neuroplasticity after ACL Injury: Implications for Optimizing Performance and Reducing Risk of Second ACL Injury. Sports Med..

[64] Deckers K., De Smedt K., Mitchell B., Vivian D., Russo M., Georgius P., Green M., Vieceli J., Eldabe S., Gulve A. (2018). New Therapy for Refractory Chronic Mechanical Low Back Pain-Restorative Neurostimulation to Activate the Lumbar Multifidus: One Year Results of a Prospective Multicenter Clinical Trial. Neuromodulation.

[65] Russo M., Deckers K., Eldabe S., Kiesel K., Gilligan C., Vieceli J., Crosby P. (2018). Muscle Control and Non-specific Chronic Low Back Pain. Neuromodulation.

[66] Freeman S., Mascia A., McGill S. (2013). Arthrogenic neuromusculature inhibition: A foundational investigation of existence in the hip joint. Clin. Biomech..

[67] Sonnery-Cottet B., Saithna A., Quelard B., Daggett M., Borade A., Ouanezar H., Thaunat M., Blakeney W.G. (2018). Arthrogenic muscle inhibition after ACL reconstruction: A scoping review of the efficacy of interventions. Br. J. Sports Med..

[68] Rice D.A., McNair P.J. (2010). Quadriceps arthrogenic muscle inhibition: Neural mechanisms and treatment perspectives. Semin. Arthritis Rheum..

[69] Taube W., Gruber M., Gollhofer A. (2008). Spinal and supraspinal adaptations associated with balance training and their functional relevance. Acta Physiol..

[70] Rudomin P., Schmidt R.F. (1999). Presynaptic inhibition in the vertebrate spinal cord revisited. Exp. Brain Res..

[71] Hopkins J.T., Ingersoll C.D. (2000). Arthrogenic muscle inhibition: A limiting factor in joint rehabilitation. J. Sport Rehabil..

[72] Becker R., Berth A., Nehring M., Awiszus F. (2004). Neuromuscular quadriceps dysfunction prior to osteoarthritis of the knee. J. Orthop. Res..

[73] Taube W., Gruber M., Beck S., Faist M., Gollhofer A., Schubert M. (2007). Cortical and spinal adaptations induced by balance training: Correlation between stance stability and corticospinal activation. Acta Physiol..

[74] Trimble M.H., Koceja D.M. (1994). Modulation of the triceps surae H-reflex with training. Int. J. Neurosci..

[75] Gruber M., Taube W., Gollhofer A., Beck S., Amtage F., Schubert M. (2007). Training-specific adaptations of H- and stretch reflexes in human soleus muscle. J. Mot. Behav..

[76] Crayton J.W., King S. (1981). Inter-individual variability of the H-reflex in normal subjects. Electromyogr. Clin. Neurophysiol..

[77] Kido A., Tanaka N., Stein R.B. (2004). Spinal excitation and inhibition decrease as humans age. Can. J. Physiol. Pharmacol..

[78] Priori A., Polidori L., Rona S., Manfredi M., Berardelli A. (2000). Spinal and cortical inhibition in Huntington’s chorea. Mov. Disord..

[79] Toft E., Sinkjaer T. (1993). H-reflex changes during contractions of the ankle extensors in spastic patients. Acta Neurol. Scand..

[80] Hodapp M., Klisch C., Mall V., Vry J., Berger W., Faist M. (2007). Modulation of soleus H-reflexes during gait in children with cerebral palsy. J. Neurophysiol..

[81] Gilbert D.L., Isaacs K.M., Augusta M., Macneil L.K., Mostofsky S.H. (2011). Motor cortex inhibition: A marker of ADHD behavior and motor development in children. Neurology.

[82] Flamand V.H., Nadeau L., Schneider C. (2012). Brain motor excitability and visuomotor coordination in 8-year-old children born very preterm. Clin. Neurophysiol..

[83] Papegaaij S., Taube W., Hogenhout M., Baudry S., Hortobagyi T. (2014). Age-related decrease in motor cortical inhibition during standing under different sensory conditions. Front. Aging Neurosci..

[84] Boorman G., Becker W.J., Morrice B.L., Lee R.G. (1992). Modulation of the soleus H-reflex during pedalling in normal humans and in patients with spinal spasticity. J. Neurol. Neurosurg. Psychiatry.

[85] Chalmers G.R., Knutzen K.M. (2002). Soleus H-reflex gain in healthy elderly and young adults when lying, standing, and balancing. J. Gerontol. A Biol. Sci. Med. Sci..

[86] Butefisch C.M., Boroojerdi B., Chen R., Battaglia F., Hallett M. (2005). Task-dependent intracortical inhibition is impaired in focal hand dystonia. Mov. Disord..

[87] McDonnell M.N., Orekhov Y., Ziemann U. (2006). The role of GABA(B) receptors in intracortical inhibition in the human motor cortex. Exp. Brain Res..

[88] Inghilleri M., Berardelli A., Cruccu G., Manfredi M. (1993). Silent period evoked by transcranial stimulation of the human cortex and cervicomedullary junction. J. Physiol..

[89] Terada M., Johnson N., Kosik K., Gribble P. (2019). Quantifying Brain White Matter Microstructure of People with Lateral Ankle Sprain. Med. Sci. Sports Exerc..

[90] Lauber B., Gollhofer A., Taube W. (2018). Differences in motor cortical control of the soleus and tibialis anterior. J. Exp. Biol..

[91] Papegaaij S., Baudry S., Negyesi J., Taube W., Hortobagyi T. (2016). Intracortical inhibition in the soleus muscle is reduced during the control of upright standing in both young and old adults. Eur. J. Appl. Physiol..

[92] Remaud A., Bilodeau M., Tremblay F. (2014). Age and muscle-dependent variations in corticospinal excitability during standing tasks. PLoS ONE.

[93] Petersen N.T., Pyndt H.S., Nielsen J.B. (2003). Investigating human motor control by transcranial magnetic stimulation. Exp. Brain Res..

[94] Simon J.E., Docherty C.L. (2018). Health-related quality of life is decreased in middle-aged adults with chronic ankle instability. J. Sci. Med. Sport.

